# The relationship between health literacy and blood sugar control in rural areas among diabetes patients

**DOI:** 10.3389/fendo.2024.1334100

**Published:** 2024-05-10

**Authors:** Jingfeng Chen, Lina Wen, Guifen Fu, Chaoqun Bai, Xiaoxue Lei, Yanping Zhang

**Affiliations:** ^1^ Department of Geriatric Endocrinology and Metabolism, Guangxi Academy of Medical Sciences and the People's Hospital of Guangxi Zhuang Autonomous Region, Nanning, China; ^2^ Cardiovascular Medicine Cadre Ward and Geriatric Cardiovascular Medicine Department, Guangxi Academy of Medical Sciences and the People's Hospital of Guangxi Zhuang Autonomous Region, Nanning, China; ^3^ Department of Nursing, Guangxi Academy of Medical Sciences and the People's Hospital of Guangxi Zhuang Autonomous Region, Nanning, China

**Keywords:** rural areas, diabetes, health literacy, blood sugar control, latent profile analysis

## Abstract

**Background:**

Although the relationship between health literacy and glycemic control has been explored in patients with diabetes, little is known about the relationship between different categories of diabetes health literacy and glycemic control in rural areas. Therefore, this study focused on the relationship between different categories of health literacy and glycemic control among diabetic patients in rural areas of Guangxi, China

**Objective:**

To explore the potential profiles of health literacy among rural diabetes patients in Guangxi and investigate their relationship with blood sugar control.

**Methods:**

A health literacy questionnaire was administered to 2280 rural diabetes patients in five cities in the Guangxi Zhuang Autonomous Region. Latent profile analysis was conducted to identify potential health literacy profiles.

**Results:**

Health literacy among rural diabetes patients in Guangxi could be categorized into high literacy-high functionality and low literacy-low criticality groups. The latent categories of health literacy were associated with blood sugar control, with diabetes patients in the high literacy-high functionality group demonstrating better blood sugar control than those in the low literacy-low criticality group *(P* < 0.05).

**Conclusion:**

Health literacy among rural diabetes patients in Guangxi exhibits heterogeneity. Healthcare professionals should pay attention to patients with low literacy and low criticality in rural areas and develop interventions to enhance their health literacy, thereby improving their blood sugar control.

## 1 Introduction

Diabetes is one of the most globally significant chronic diseases, with complex pathophysiology and unclear etiology. According to a report from the International Diabetes Federation (IDF), China has the highest number of diabetes patients in the world, with nearly 114 million adult cases, accounting for 24% of the total global diabetes population (1). Furthermore, the growth of diabetes patients in rural areas has outpaced urban areas (2). This increase in prevalence has placed a considerable burden on healthcare systems and management costs (3). Surveys indicate that the prevalence of diabetes in adults in Guangxi’s rural areas is 5.9%, with only 22.2% achieving adequate blood sugar control (4). Diabetes primarily results from insufficient insulin secretion or resistance, and poor long-term blood sugar control can lead to various complications. Therefore, maintaining good blood sugar control is essential for preventing and delaying complications (5).

Currently, diabetes cannot be cured, and patients require lifelong oral antidiabetic medication or subcutaneous insulin injections to effectively control blood sugar, slow disease progression, reduce complication rates, and improve prognosis (6).Health literacy refers to an individual’s ability to obtain health information through various channels, correctly understand and apply this information to actively maintain and promote their own health (7). The American Diabetes Association includes improving health literacy among diabetes patients as an essential component of overall diabetes management in its “Standards of Medical Care in Diabetes” (8). Diabetes patients with lower health literacy, due to limited reading and mathematical abilities, are more likely to have suboptimal blood sugar control and an increased risk of diabetes complications (9). Conversely, a higher level of health literacy can effectively control blood sugar levels, manage complications, and enhance quality of life for diabetes patients (10). The level of health literacy may vary among different diabetes patients based on factors such as cognitive level and treatment stage (11). Analyzing health literacy levels and capturing individual characteristics are prerequisites for implementing personalized and precise interventions.

Latent profile analysis is a “person-centered” statistical method that classifies heterogeneous populations into different homogeneous subgroups based on health literacy levels. This approach offers new insights for individualized and precise interventions. Currently, research on the health literacy of diabetes patients, both domestically and internationally, tends to focus on variable-centered research methods, with limited studies on category divisions and the relationship between different categories and blood sugar control. This study utilizes latent profile analysis to categorize the health literacy of diabetes patients in rural areas and analyze the relationship between different categories and blood sugar control. This research aims to provide a basis for diabetes health management in rural areas in the future.

## 2 Materials and methods

### 2.1 Sample

Between January 2022 and July 2022, a multi-stage stratified sampling method was employed to select the study subjects. In the first stage, five cities, namely Nanning, Guilin, Hechi, Chongzuo, and Yulin, were randomly selected based on the geographical regions of the Guangxi Zhuang Autonomous Region (central, eastern, southern, western, and northern). In the second stage, three counties were randomly chosen within each of the selected cities, resulting in a total of 15 counties for the survey. In each county, one internal medicine department from a local hospital was selected for the investigation. A total of 152 individuals were surveyed in each hospital, resulting in 2280 patients being surveyed in total.Inclusion criteria were as follows: (1) Conforming to the diagnostic criteria for diabetes established by the Chinese Diabetes Society in 2020 (8); (2) Age ≥ 18 years; (3) Registered and currently residing in the surveyed area. Exclusion criteria were as follows: (1) Patients with gestational diabetes; (2) Patients with a known history of dementia or other mental disorders based on medical records or information obtained from family members; (3) Patients in the acute phase of their illness, unable to cooperate with the investigation. Written informed consent was obtained from all participants, and informed consent was provided by the administrators of all hospitals participating in this study.

### 2.2 Survey tools

#### 2.2.1 General information questionnaire

This questionnaire was developed by the researchers and includes demographic information such as age, gender, marital status, educational level, and per capita annual disposable income (9). It also covers disease-related data, including disease duration, glycated hemoglobin levels, whether the participant is using antidiabetic medication, and whether they are undergoing insulin treatment.

#### 2.2.3 Health literacy scale

The Health Literacy Scale (HLS) is an assessment tool developed by Ishikawa et al. in 2008 for evaluating the health literacy of diabetes patients. In 2021, Zhao Xiaoyan et al. (12) translated and validated the scale into Chinese. The scale consists of three dimensions: functional health literacy, communicative health literacy, and critical health literacy, encompassing a total of 14 items. Respondents use a 4-point Likert scale, with scores ranging from 1 to 4, corresponding to “completely absent” to “most of the time.” Notably, the functional health literacy dimension is reverse-scored. The total score on the scale ranges from 14 to 56, with higher scores indicating better health literacy. The Cronbach’s α coefficient for the Chinese version of HLS is 0.868.

### 2.3 Ethical considerations

The hospital where the authors are affiliated received approval from the Ethics Committee (Approval Number: KT-KJT-2021-26). The questionnaire outlined the voluntary and confidential nature of participation, and all hospital administrators and participants provided informed consent.

### 2.4 Data collection

This study involved questionnaire surveys, blood collection at local hospitals, and assisting patients in filling out standardized questionnaires. Researchers recorded general information and glycated hemoglobin values for diabetes patients in 15 hospitals and entered the results into Excel, which were then transmitted to us via the internet. Upon completion of the questionnaires, they were also sent to us electronically. We compiled data from the 15 hospitals and stored it in Excel spreadsheets. All data were independently verified and input into SPSS by two researchers. All information is stored on a secure computer to prevent data leakage.

### 2.5 Data analysis

This study employed SPSS 22.0 and Mplus 8.3 software for data statistical analysis and processing. (1) The health literacy scores of rural diabetes patients, treated as a continuous variable, were used as the manifest variable for latent profile data processing with Mplus 8.3 (13). The initial model started from “1” and gradually increased the number of latent categories until the best model fit was achieved. Fit indices included: (i) Akaike Information Criteria (AIC), Bayesian Information Criteria (BIC), and adjusted Bayesian Information Criteria (aBIC), with smaller values indicating better fit; (ii) Entropy, which represents classification accuracy, and when Entropy > 0.8, it indicates classification accuracy > 90%; (iii) Model comparisons were primarily based on the Bootstrap Likelihood Ratio Test (BLRT) and Likelihood Ratio Test (LMR). When the p-values corresponding to LMR and BLRT reached a significance level, it indicated that the model had a better fit than the previous one. This study comprehensively evaluated the fit of various category models and selected the best model.(2) Data were analyzed using SPSS 25.0 software. For quantitative data that followed a normal distribution, descriptive statistics were presented as x ± s (mean ± standard deviation). For data that did not follow a normal distribution, descriptive statistics were presented as M (Q1, Q3) (median with interquartile range). Qualitative data were described using frequency counts. Multivariate logistic regression analysis was used to determine whether statistically significant differences existed among different latent profile groups concerning their manifest characteristics. Based on these results and considering the normal distribution of the total health literacy scores, different latent profile groups’ health literacy levels were compared using t-tests, one-way analysis of variance, or non-parametric tests such as the Mann-Whitney U test and Kruskal-Wallis H test. All tests were two-tailed, and *p*<0.05 was considered statistically significant.

## 3 Results

### 3.1 Basic information of patients

A survey was conducted on 2,280 rural diabetic patients, of which a total of 2,178 rural diabetic patients were effectively surveyed. The basic profile of the patients surveyed is shown in **Table 1**. The surveyed rural diabetic patients had an average age of (63.25 ± 12.71) years and an average duration of illness of (7.96 ± 4.07) years. The male proportion was 55.28%, indicating a higher representation of males than females. In terms of educational background, the majority had completed junior high school education, accounting for 31.43%. The proportion of patients with per capita disposable annual family income ≥18931 yuan was 37.74%. The proportion of patients with a family history of diabetes was 14.55%, and the proportion of patients with HbA1c control <7.0% was 22.68%.

**Table 1:** General information of surveyed rural diabetic patients (n=2178).

Variable	Group	Count(Percentage,%)
Gender	Male	1204(55.28)
	Female	974(44.72)
Marital Status	Married	2020(92.75)
	Unmarried or Widowed	158(7.25)
Education Level	Illiterate	256(11.75)
	Primary School	504(23.14)
	Junior High School	684(31.41)
	High School	507(23.28)
	Associate Degree	180(8.26)
	Bachelor’s Degree	47(2.16)
Average Family Disposable	<18931 Yuan	1356(62.26)
	≥18931Yuan	822(37.74)
Family History of Diabetes	Yes	317(14.55)
	No	1861(85.45)
HbA1c Control Status	<7.0	494(22.68)
	≥7.0	1684(77.32)

### 3.2 Latent profile analysis of health literacy in rural diabetes patients

The fitting statistics for the profile models are shown in **Table 2**. According to the entropy, the class 2 model fits the best. And the P-values of both LMR and BLRT were statistically significant, so the class 2 model was chosen as the final model.

**Table 2:** Model fit indices of latent profile models for health literacy in rural diabetes patients (n=2178).

Category	AIC	BIC	aBIC	Entropy	LMP(*P*)	BLRT(*P*)	Category Probability
1	35161.222	35195.339	35176.276	–	–	–	1
2	31182.114	31238.975	31207.204	0.976	<0.001	<0.001	0.54、0.46
3	30439.823	30519.429	30474.949	0.970	<0.001	<0.001	0.21、0.46, 0.33
4	29941.294	30043.645	29986.457	0.929	<0.001	<0.001	0.10、0.33、 0.36, 0.21
5	29298.183	29423.279	29353.382	0.949	<0.001	<0.001	0.21、0.33、0.07、0.19、0.20

### 3.3 Characteristics and naming of latent casses for health literacy in rural diabetes patients

The health literacy of diabetic patients in rural areas is categorized into two potential groups, showing distinct characteristics across three dimensions. Category 1, with a total health literacy score of (39.53 ± 4.795), exhibits relatively higher levels of health literacy across all dimensions. Among these, the functional health literacy dimension scored the highest at (14.72 ± 1.366), thus labeled as the high literacy-high functionality group. Category 2, with a total health literacy score of (22.82 ± 3.768), demonstrated lower scores across various dimensions of health literacy. Particularly, the critical dimension scored the lowest at (6.24 ± 1.280), hence named the low literacy-low criticality group. The scores for each dimension of health literacy for the two categories of rural diabetic patients are detailed in **Table 3**.

**Table 3:** Health literacy scores for different categories of rural diabetes patients (n=2178).

Groups	Total health literacy score	Functional health literacy	Communicative health literacy	Critical health literacy
High Literacy - High Functional(n=997)	39.53±4.795	14.72±1.366	13.30±3.405	11.51±1.900
Low Literacy - Low Critical(n=1181)	22.82±3.768	8.17±2.202	8.40±2.767	6.24±1.280
General level	30.47±9.357	11.17±3.757	10.64±3.926	8.65±3.070

### 3.4 Comparison of socio-demographic characteristics of different latent classes of health literacy in rural diabetes patients

Comparison of socio-demographic characteristics of different potential categories of health literacy among diabetic patients in rural areas is shown in **Table 4**, which shows that the differences between the two groups were statistically significant (p < 0. 05) in terms of educational level (χ^2^ = 83.169, *p* < 0. 001), per capita annual disposable income of the family (χ^2^ = 64.792, *p* < 0. 001), the duration of the disease with or without complications (χ^2^ = 10.106, *p*<0.001), family history of diabetes (χ^2^ = 27.362, *p*<0.001) the difference was statistically significant (*p*<0. 05).

**Table 4:** Comparison of socio-demographic characteristics of different latent classes.

Projects	High Literacy - High Functional (n=997)	Low Literacy - Low Critical (n=1181)	Statistical value	*p*
Age	63.79±12.670	62.80±12.734	1.807a	0.071
Genders			1.497^b^	0.221
Man	537	667		
Females	460	514		
Education level			83.169^b^	<0.001
Illiteracy	63	193		
Elementary school	212	292		
Middle school	317	367		
High school	264	243		
Specialized education	108	72		
Undergraduate	33	14		
Marital status			1.100^b^	0.294
Married	931	1089		
Unmarried or widowed	66	92		
Per capita annual disposable income			64.792^b^	<0.001
<18931 yuan	530	826		
≥18931yuan	467	355		
Complications			10.106^b^	<0.001
Yes	686	885		
No	311	296		
Family history of diabetes			27.362^b^	<0.001
Yes	188	129		
No	809	1052		

a: t-test; b: χ2 test.

### 3.5 Comparison of HbA1c control rate in rural diabetes patients with different latent profiles of health literacy

The standardized HbA1c rate for all study subjects was 22.68%. The high literacy-high functionality group demonstrated a blood sugar control rate of 39.12% (390/997), while the low literacy-low criticality group exhibited a blood sugar control rate of 9.66% (104/1077). The blood sugar control rate in the high literacy-high functionality group was significantly higher than that in the low literacy-low criticality group (*P* < 0.001). The results are presented in **Table 5**.

**Table 5:** Comparison of HbA1c control rate in DM patients with different health literacy levels.

Groups	HbA1c≥7.0	HbA1c<7.0	*χ^2^*	*p*
High Literacy - High Functional	607	390	283.232	<0.001
Low Literacy - Low Critical	1077	104		
Total	1684	494		

### 3.6 Impact of health literacy level in diabetes patients on blood sugar control

The HbA1c standardization rate was considered as the dependent variable, while health literacy served as the independent variable. After controlling for variables such as age, gender, educational level, family per capita disposable income, family history of diabetes, medication usage, insulin use, and presence of comorbidities, a variable screening using the entry method (α = 0.05) was conducted for logistic regression analysis. The results showed that age, gender, presence of medication, insulin use, education level, per capita disposable household income, and health literacy category were the influencing factors of HbA1c attainment rate, (p<0.05),The attainment rate of HbA1c control was higher in males as compared to females (OR:0.764,CI:0.606-0.964). Patients in the high-literacy-high-functionality group may have a higher rate of achieving HbA1c control compared to patients in the low-literacy-low-criticism group (OR: 5.295, CI 4.103-6.827). Patients taking oral hypoglycaemic agents had a higher rate of achieving HbA1c control compared to patients not taking oral hypoglycaemic agents (OR: 2.270, CI: 1.750-2.943). Patients using insulin had a higher rate of achieving HbA1c control compared to patients not using insulin (OR: 2.957, CI:2.338-3.740). Compared with patients with per capita disposable annual household income <18931 yuan, patients with per capita disposable annual household income ≥18931 yuan had a higher rate of achieving HbA1c control (OR: 1.682, CI: 1.331-2.125). The results are presented in **Table 6**.

**Table 6:** Logistic regression analysis of HbA1c control rate in rural diabetes patients.

Variable	β	SE	*Waldχ^2^*	*p*	OR(95%CI)
Constant	-3.104	0.500	38.537	<0.001	–
Health Literacy Category					
High Literacy - High Functional	1.667	0.130	165.168	<0.001	5.295(4.103,6.827)
Low Literacy - Low Critical	–	–	–	–	–
Age	0.026	0.005	28.236	<0.001	1.026(1.016,1.036)
Genders					
Man	-0.269	0.119	5.131	0.024	0.764(0.606,0.964)
Female	–	–	–	–	–
Family history of diabetes					
Yes	0.150	0.155	0.936	0.333	1.162(0.858, 1.574)
No	–	–	–	–	–
Oral hypoglycemic therapy					
Yes	0.820	0.133	38.211	<0.001	2.270(1.750,2.943)
No	–	–	–	–	–
Treatment with insulin					
Yes	1.084	0.120	81.884	<0.001	2.957(2.338,3.740)
No	–	–	–	–	–
Education level			36.213	<0.001	
Illiteracy	–	–	–	–	–
Elementary school	1.416	0.399	12.583	<0.001	4.122(1.885,9.016)
Middle school	1.499	0.361	17.205	<0.001	4.477(2.205,9.091)
High school	0.825	0.347	5.643	0.018	2.283(1.155,4.510)
Specialized education	0.780	0.351	4.946	0.026	2.182(1.097,4.341)
Undergraduate	0.545	0.375	2.109	0.46	1.725(0.826,3.599)
Per capita annual disposable income					
<18931 yuan	–	–	–	–	–
≥18931yuan	0.520	0.119	18.997	<0.001	1.682(1.331,2.125)
Complications					
Yes	-0.004	0.130	0.001	0.977	0.996(0.773,1.284)
No	–	–	–	–	–

## 4 Discussion

### 4.1 Characteristics of different latent profiles of health literacy in rural diabetes patients

The use of latent profile analysis aids in identifying heterogeneity among rural diabetes patients, facilitating the identification of variations in health literacy levels among individuals. This approach helps pinpoint issues related to health literacy among patients and allows for targeted interventions aimed at improving their knowledge level, thereby serving as a reference for interventions in glycemic control. This study employed latent profile analysis to classify rural diabetes patients into two latent categories based on health literacy: the high literacy-high functionality group and the low literacy-low criticality group. The latter, constituting 54.22% (1181/2178) of the total, exhibits relatively lower scores across health literacy dimensions compared to the overall level. This suggests difficulties in accessing and utilizing health information and inaccurate judgment of health information among these patients. Probable causes include the relatively underdeveloped economic status of Guangxi, situated in the western region of China (12), with rural diabetes patients having lower educational levels and relatively lower incomes (14, 15). Consequently, these patients have limited disease-related knowledge and information acquisition abilities, compounded by long disease durations leading to decreased disease awareness (16). Healthcare providers should encourage these patients to acquire knowledge through various channels and offer personalized or group health education to enhance their understanding and acquisition of health information, especially for those with lower literacy and limited learning abilities. The high literacy-high functionality group comprises 45.78% (997/2178) of the patients, displaying higher scores in all dimensions of health literacy compared to the overall level. Notably, this group excels particularly in the functional aspect of health literacy, indicating their willingness to invest time and effort in understanding diabetes-related health knowledge. This may be attributed to their relatively higher cultural and knowledge levels, enhancing their reading and information utilization abilities. Healthcare providers should encourage patients in this group to foster a strong desire to improve health and enhance their awareness of health.

### 4.2 Factors affecting health literacy in rural diabetes patients

By exploring the differences in disease-related information and demographic characteristics among different health literacy categories, it was found that patients in the high literacy-high functionality group have higher educational levels and relatively younger ages compared to those in the low literacy-low criticality group, which is consistent with other studies (17–19). Additionally, the low literacy-low criticality group of diabetes patients showed a higher prevalence of comorbid complications, suggesting an inverse relationship between the occurrence of diabetes complications and the level of health literacy. Ajuwon’s findings show that lower health literacy among African Americans is associated with lower self-management behaviours that can lead to complications of type 2 diabetes (20). This is similar to our findings. The results of this study also indicate that patients in the high literacy-high functionality group have higher average monthly family incomes compared to those in the low literacy-low criticality group, consistent with other research (21, 22). This could be attributed to an increased awareness of disease treatment in situations where material needs are met, prompting patients to actively invest in their health, thereby enhancing their health literacy regarding chronic diseases.

### 4.3 Relationship between latent profiles of health literacy and blood sugar control in rural diabetes patients

Studies (23–25) have indicated a significant negative correlation between health literacy among diabetes patients and glycemic control. In this study, the high literacy-high functionality group exhibited the highest number of patients meeting blood sugar control standards, while the low literacy-low criticality group showed fewer patients meeting these standards, highlighting differences in glycemic control between the two categories of patients. Logistic regression analysis revealed that the diverse latent profiles of health literacy among rural diabetes patients are influencing factors in glycemic control. Patients in the high literacy-high functionality group exhibited significantly better glycemic control compared to those in the low literacy-low criticality group. Achieving glycemic control reflects an individual’s spontaneous and multi-dimensional health-promoting behavior aimed at maintaining or improving their own health status (26). A higher level of health literacy in diabetes patients is associated with a better understanding of disease-related knowledge, improved treatment adherence, and a higher level of glycemic control (27). Therefore, healthcare professionals can encourage patients to learn and acquire knowledge related to diabetes. For diabetes patients in remote rural areas of Guangxi, interventions such as establishing platforms for diabetes knowledge acquisition and exchange could be implemented to enhance patient health literacy, consequently elevating their level of glycemic control.

## 5 Limitations

This was a multicenter cross-sectional study, and we conducted questionnaire surveys in 15 county-level hospitals. While we selected rural diabetes patients according to national diagnostic criteria, the models of the hemoglobin A1c measurement devices in these 15 county hospitals were not identical, resulting in slight variations in test results between hospitals. We were unable to transport these devices to rural areas for the study, so we chose to survey rural diabetes patients who sought treatment at the hospitals. Therefore, caution should be exercised when applying these findings for broader use. Second, we assessed patient literacy using the Health Literacy Scale, a tool that can be time-consuming in clinical use.

## 6 Conclusion

There are variations in health literacy levels among rural diabetes patients, categorizing them into two groups: high literacy with high functionality and low literacy with low criticality. The proportion of individuals with high literacy is relatively small, indicating significant room for improvement. Moreover, the group with low literacy and low criticality has a lower rate of blood glucose control. Healthcare professionals can enhance the health literacy of rural diabetes patients to improve their blood glucose control outcomes.

## Data availability statement

The original contributions presented in the study are included in the article/supplementary material. Further inquiries can be directed to the corresponding author.

## Ethics statement

The studies involving humans were approved by Ethics Committee of the People’s Hospital of Guangxi Zhuang Autonomous Region. The studies were conducted in accordance with the local legislation and institutional requirements. The participants provided their written informed consent to participate in this study.

## Author contributions

JC: Writing – original draft, Data curation. LW: Writing – review & editing. GF: Writing – review & editing, Data curation. CB: Writing – original draft, Investigation, Data curation. XL: Writing – original draft, Investigation, Data curation. YZ: Writing – review & editing, Project administration.
